# Whole-Transcriptome Profiling of Ovarian Tissues in Gilts with Normal Estrus and Follicular Cyst-Associated Anestrus

**DOI:** 10.3390/ijms27156848

**Published:** 2026-07-30

**Authors:** Lingyan Lv, Jiaqing Zhang, Xianhua Wu, Changhua Lin, Yangzu Zhang, Hongfang Mo, Jiapeng Li, Xun Li, Jiaming Zheng, Chuanhuo Hu

**Affiliations:** 1 College of Animal Science and Technology, Guangxi Key Laboratory of Animal Breeding, Disease Control and Prevention, Guangxi University, Nanning 530004, China; 2 Institute of Animal Husbandry, Henan Academy of Agricultural Sciences, Zhengzhou 450002, China

**Keywords:** gilts, ovary, whole transcriptome sequencing, normal estrus, follicular cyst-associated anestrus, ceRNA regulatory network

## Abstract

We explored the potential functions of differentially expressed miRNAs, mRNAs, lncRNAs, and circRNAs identified in the ovaries of gilts with normal estrus (NE) and follicular cyst-associated anestrus (AE), as well as their putative competing endogenous RNA (ceRNA) regulatory networks. Ovarian morphology was assessed via ultrasonography, and serum concentrations of Follicle-stimulating hormone (FSH), (Estradiol) E2, and Progesterone(P4) were measured. Ovarian tissues were harvested after slaughter for whole-transcriptome sequencing. Bioinformatic tools were used to screen differentially expressed RNAs(DERNAs) between NE and AE gilts. We further predicted target interactions among these transcripts, conducted functional enrichment analysis on target genes, and constructed candidate ceRNA regulatory networks potentially associated with gilt estrus. Phenotypic verification confirmed that ovarian ultrasonographic characteristics, histological morphology, and serum reproductive hormone levels were consistent with the physiological status of NE and AE gilts. Under the screening thresholds of *p* < 0.05 and |log_2_FC| ≥ 1, we identified 22 lncRNAs that may interact with 21 mRNAs via 50 miRNAs, alongside 39 circRNAs predicted to regulate 26 mRNAs through 72 miRNAs. Functional enrichment analysis indicated that target genes of these differentially expressed transcripts were predominantly enriched in the lysosome pathway, PPAR signaling pathway, chemokine signaling pathway, cholesterol metabolism and NOD-like receptor signaling pathway. Hub molecules including *FGF1*, *GHR*, *TLR2*, ssc-miR-370, and miR-21-5p were shared in lncRNA/circRNA-miRNA-mRNA regulatory networks; these molecules have been reported to participate in progesterone synthesis, estrus modulation, and endocrine homeostasis. Of particular interest, two non-coding RNAs, MSTRG.1285.1 and novel_circ_056113, were predicted to act as candidate ceRNAs that may sponge ssc-miR-370 and miR-21-5p, which could in turn modulate the expression of estrus-associated mRNAs including *FGF1*, *GHR*, and *TLR2*. The expression trends of MSTRG.1285.1, novel_circ_056113, miR-370, miR-21-5p, *GHR*, *TLR2*, and *FGF1* were validated by qRT-PCR, and the quantification results agreed with transcriptome sequencing data. Collectively, this study constructed a predicted ceRNA regulatory network of ovarian transcripts comparing NE and AE gilts and uncovered multiple RNA molecules potentially involved in estrus regulation. These findings offer preliminary theoretical clues for exploring the onset of puberty in gilts.

## 1. Introduction

The ovary is the most vital reproductive organ in female mammals. It mainly produces developmentally totipotent oocytes. It also secretes steroid hormones [1], regulating estrous cycle activities [2,3]. Arguably, it is involved in nearly all aspects of the reproductive process in sows. Therefore, ovarian function is the key factor influencing sow reproductive performance.

Currently, the ceRNA research idea has been applied to studies examining differences in the longissimus dorsi muscle of various pig breeds, differences in porcine skeletal muscle, and pork fat deposition [4,5,6]. However, there is a scarcity of relevant research; in fact, the estrus process in sows is governed by a complex and tightly regulated molecular network, which is collectively influenced by numerous genes and transcription factors, with non-coding RNAs playing a vital role in this regulation [7]. These non-coding RNAs include long non-coding RNAs (lncRNAs), microRNAs (miRNAs), circular RNAs (circRNAs), and others [8]. Salmena et al. [9] introduced the “competing endogenous RNA (ceRNA)” hypothesis, describing a molecular mechanism where non-coding RNAs and protein-coding genes competitively bind miRNAs via miRNA response elements (MREs). Both lncRNAs [10] and circRNAs [11] can serve as miRNA sponges, absorbing miRNAs and functioning as ceRNAs, thereby affecting miRNA activity, regulating mRNA expression, and ultimately influencing various biological processes [12,13]. CeRNA regulatory networks participate in multiple biological processes, especially reproduction and development. Previous studies have reported their functions in regulating gonadal development in golden pompano [14], as well as reproductive performance in Hy-Line Brown laying hens [15] and goats (*Capra hircus*) [16]. Accumulating evidence also reveals that ceRNA pathways mediate estrus regulation in ovarian tissues [17]. However, few studies are focusing on the role of ceRNAs in gilt estrus traits; therefore, it is necessary to identify key mRNAs and non-coding RNAs that regulate the estrous cycle in gilts, thereby establishing a theoretical foundation for a deeper understanding of how these RNAs mediate target genes to regulate estrus. 

Abnormal estrus, including anestrus (silent estrus representing a form of apparent anestrus) and persistent estrus, arises from disruption of the hypothalamic-pituitary-ovarian endocrine cascade [18]. Deficiencies in the preovulatory luteinizing hormone surge prevent normal ovulation, leading to persistent anovulatory follicles and follicular cyst formation in gilts [19,20]. Disturbed steroid secretion further impairs behavioral estrus signals, generating silent estrus: functional ovulation occurs without visible estrous manifestations [21]. Clinically, these disorders extend non-productive days, decrease farrowing rates, and increase herd elimination costs, posing substantial threats to swine reproductive efficiency [22]. The high rearing costs of gilts and the occurrence of anestrus are primary reasons for their large-scale culling, leading to substantial economic losses. Therefore, this study conducted whole-transcriptome sequencing of ovarian tissues from NE gilts and AE gilts with follicular cyst-associated silent estrus to establish a ceRNA regulatory network. This study aimed to screen key mRNAs and non-coding RNAs associated with the regulation of silent estrus by follicular cysts in gilts, laying a preliminary transcriptomic reference for future research on ovarian estrous regulatory networks.

## 2. Results

### 2.1. Ovarian Morphological Characteristics and Serum Reproductive Hormone Levels of NE/AE Gilts 

**Figure 1:**
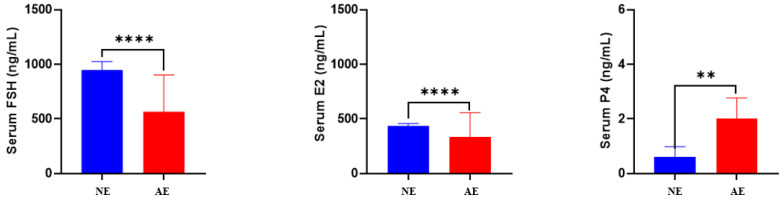
Serum levels of reproductive hormones in NE and AE gilts. Note: Values are presented as mean± SD. An independent samples *t*-test was used for intergroup comparison. ** represents *p* ≤ 0.01, **** represents *p* < 0.0001.

**Figure 2:**
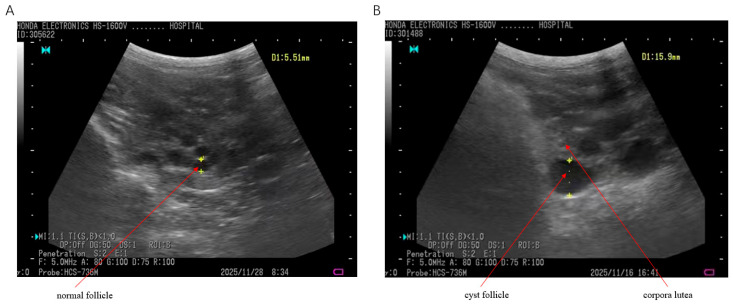
Ultrasonographic morphology of ovarian tissue in gilts; the arrows point to follicles and corpora lutea (The corpus luteum features solid parenchymal tissue inside and manifests as a regular circular gray area under ovarian ultrasonography); The yellow-marked regions in Figure 2A represent normally developing follicles, while those in Figure 2B represent cystic follicles. (**A**) Normal ovary; (**B**) Follicular cyst ovary.

**Figure 3:**
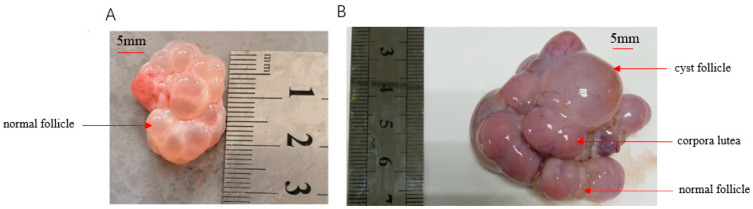
Morphology of ovarian tissue in gilts. (**A**) Normal ovary; (**B**) Follicular cyst ovary.

Serum concentrations of FSH and E2 were significantly lower in AE gilts compared with NE gilts, whereas serum P4 concentration was markedly increased in AE gilts (FSH: NE-947.98 ± 26.04 ng/mL, AE-565.75 ± 111.84 ng/mL; E2:NE-435.03 ± 7.46 ng/mL, AE 335.20 ± 73.60 ng/mL; P4: NE-0.61 ± 0.13 ng/mL, AE-2.01 ± 0.25 ng/mL, Figure 1). Distinct ovarian morphological differences between the two groups were revealed by ultrasonography: NE ovaries (Figure 2A) showed homogeneous parenchymal echogenicity with clear follicular structures (follicle diameter = 5.51 mm), whereas AE ovaries (Figure 2B) displayed large, well-defined anechoic cysts (diameter = 15.9 mm), indicative of ovarian cyst formation. Consistent with these hormonal differences, the gross anatomical features of ovaries differed markedly between the two groups. Ovaries from NE gilts were covered with numerous evenly sized, small-to-medium translucent normal follicles (Figure 3A). In contrast, ovaries from AE gilts featured prominent functional corpora lutea alongside one large abnormal follicle (follicular cyst), with only a small number of minor normal follicles retained (Figure 3B). The presence of intact corpora lutea indicates that these AE gilts had successfully ovulated before follicular cyst formation, which may suggest their reproductive disorder manifests as silent estrus rather than anovulatory complete anestrus. 

### 2.2. Transcriptomic Profiles of miRNAs, mRNAs, lncRNAs and circRNAs in NE/AE Gilt Ovaries 

**Figure 4:**
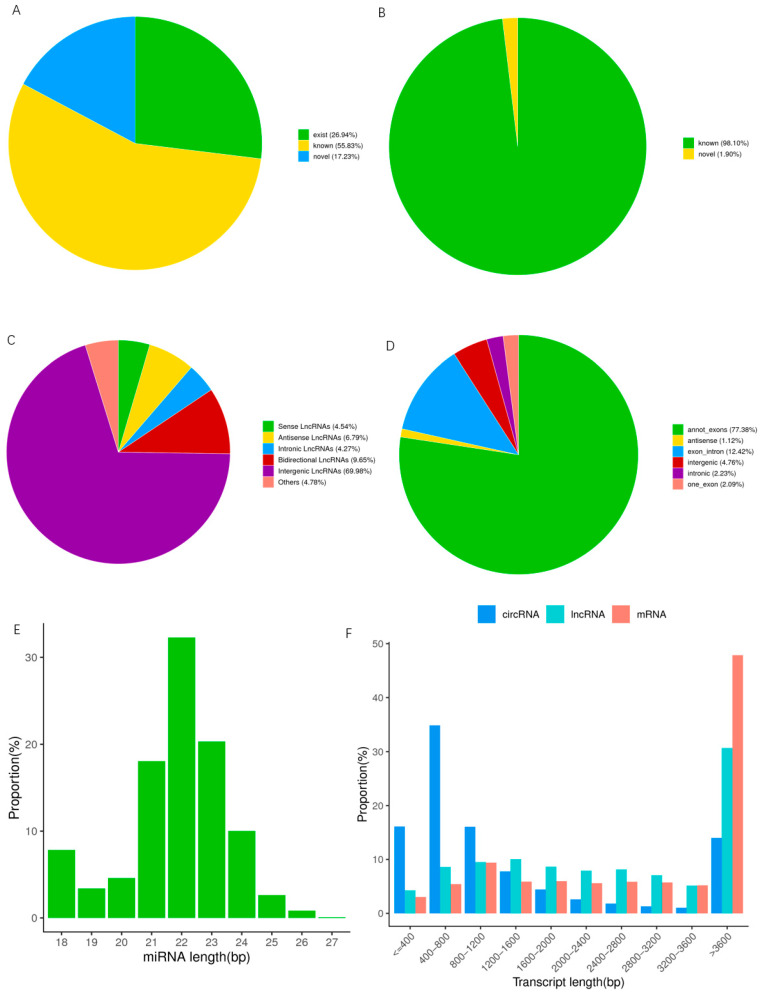

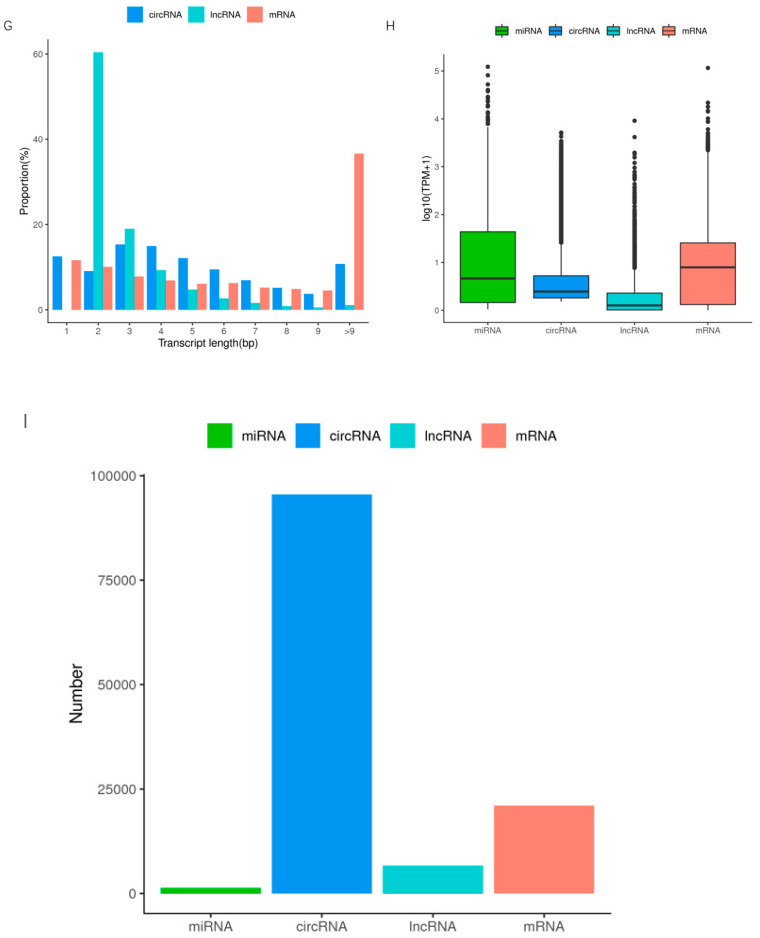
Characterization and comparison of miRNAs, mRNAs, lncRNAs, and circRNAs in ovarian tissues of NE and AE gilts. (**A**–**D**) Classification and distribution of the four types of RNAs. (**E**,**F**) Length distribution of miRNAs, mRNAs, lncRNAs, and circRNAs. (**G**) Exon number distribution of mRNAs, lncRNAs, and circRNAs. (**H**,**I**) Comparison of expression levels of the four RNA types between groups.

A total of twelve sequencing libraries were constructed, including six small RNA libraries for miRNA profiling and six strand-specific whole-transcriptome libraries for the detection of lncRNAs, circRNAs and mRNAs. Detailed statistics regarding sequencing-data filtering, base-quality profiling, read alignment, tag-abundance analysis, database comparison, and miRNA identification are summarized in Tables S1–S11. Distinct compositional profiles across RNA biotypes were observed:

(1) In the miRNA libraries, known types accounted for the highest proportion (55.83%), followed by existing types (26.94%), while novel types were the least abundant (17.23%).
(2) In the mRNA libraries, the vast majority (98.10%) of transcripts were classified as known types, with only 1.90% identified as novel.
(3) For lncRNAs, intergenic LncRNAs constituted the largest category (69.98%), followed by bidirectional LncRNAs (9.65%), antisense LncRNAs (6.73%), sense LncRNAs (4.54%), intronic LncRNAs (4.27%), and other remaining types (4.78%).
(4) CircRNAs were primarily derived from exonic regions (77.36%), with smaller proportions from exon_intron (12.42%), intergenic (4.76%), intronic (2.23%), antisense (1.12%), and one_exon (2.09%) sources (Figure 4A–D).

Using transcript length distribution analysis, it was revealed that over 95% of miRNA sequences ranged from 20 to 24 nt, with 22 nt being the most abundant size class-consistent with the canonical characteristics of mature miRNAs (Figure 4E). For other RNA types, most lncRNAs were longer than 800 bp, circRNAs were predominantly 400–800 bp in length, and mRNAs exhibited a broad size distribution spanning from ≤400 bp to >3600 bp (Figure 4F). After exon number analysis, it was shown that most lncRNAs contained only 2–3 exons, whereas mRNAs typically had 9 or more exons (Figure 4G). Finally, through the comparison of expression levels, it was indicated that mRNAs generally exhibited higher expression than lncRNAs (Figure 4H,I).

### 2.3. Transcriptome-Wide Characterization of miRNAs, mRNAs, lncRNAs, and circRNAs in NE/AE Gilt Ovaries

**Figure 5:**
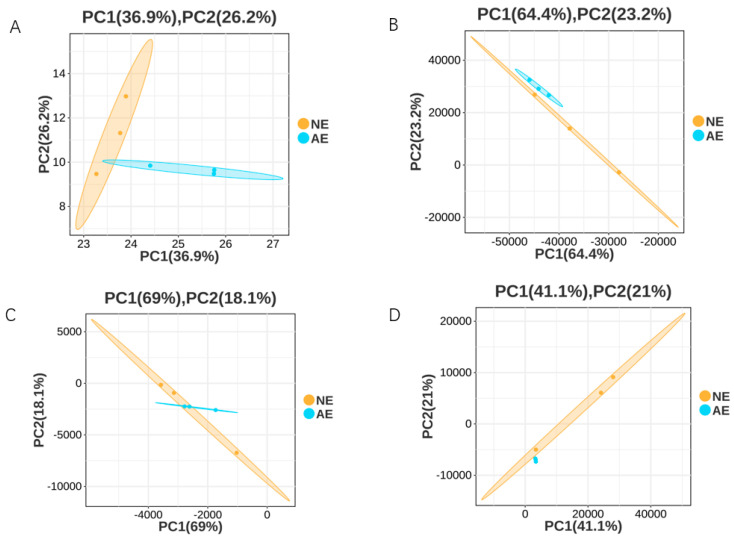
Principal component analysis (PCA) of miRNA, mRNA, lncRNA, and circRNA expression profiles in ovarian tissues of NE and AE gilts. (**A**) miRNAs; (**B**) mRNAs; (**C**) lncRNAs; (**D**) circRNAs.

Principal component analysis (PCA) was conducted to evaluate the similarities and differences in RNA expression profiles between ovarian tissues from gilts under NE and AE conditions. As presented in Figure 5, biological replicates from the same group clustered tightly together, confirming good reproducibility of the transcriptome sequencing data. Obvious separation was detected between the two physiological groups, which indicated that the expression patterns of miRNAs, mRNAs, lncRNAs, and circRNAs differed markedly between NE and AE gilts. Collectively, through these findings, it was revealed that the estrus status strongly modulated the expression of various RNA molecules, which supported further differential expression analysis.

**Figure 6:**
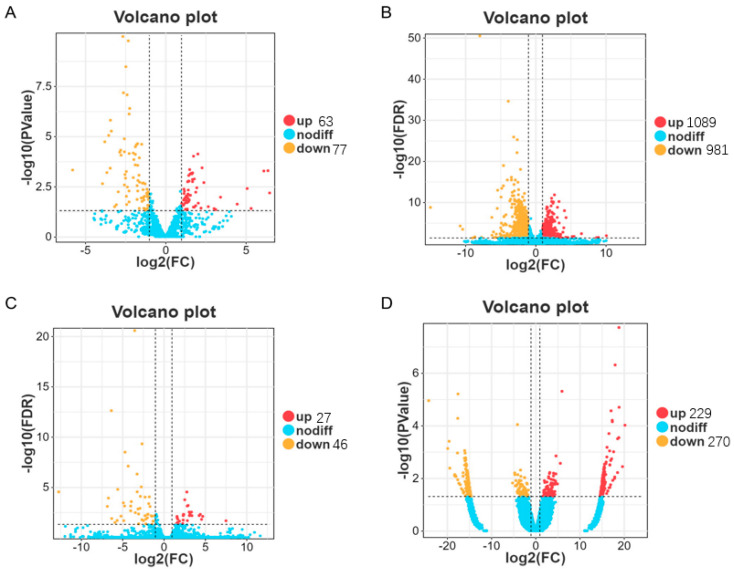
Volcano plots of DERNAs in ovarian tissues of NE and AE gilts, Vertical dotted lines indicate the threshold of |log_2_(FC)|, and the horizontal dotted line denotes the significance threshold for *p*-value or FDR. (**A**) DEmiRNAs; (**B**) DEmRNAs; (**C**) DElncRNAs; (**D**) DEcircRNAs.

Volcano plots were then constructed to screen DERNAs between the NE and AE groups (Figure 6). With the thresholds of FDR < 0.05 and |log_2_FC| ≥ 1, a total of 63 upregulated and 77 downregulated DEmiRNAs, 1089 upregulated and 981 downregulated DEmRNAs, 27 upregulated and 46 downregulated DElncRNAs, as well as 229 upregulated and 270 downregulated DEcircRNAs were identified. The volcano plots visually displayed the distribution of upregulated, downregulated, and non-differentially expressed transcripts, demonstrating extensive transcriptomic differences in gilt ovaries associated with different physiological states.

### 2.4. Hierarchical Clustering Heatmap of DEmiRNAs, mRNAs, lncRNAs, and circRNAs in NE/AE Gilt Ovaries

**Figure 7:**
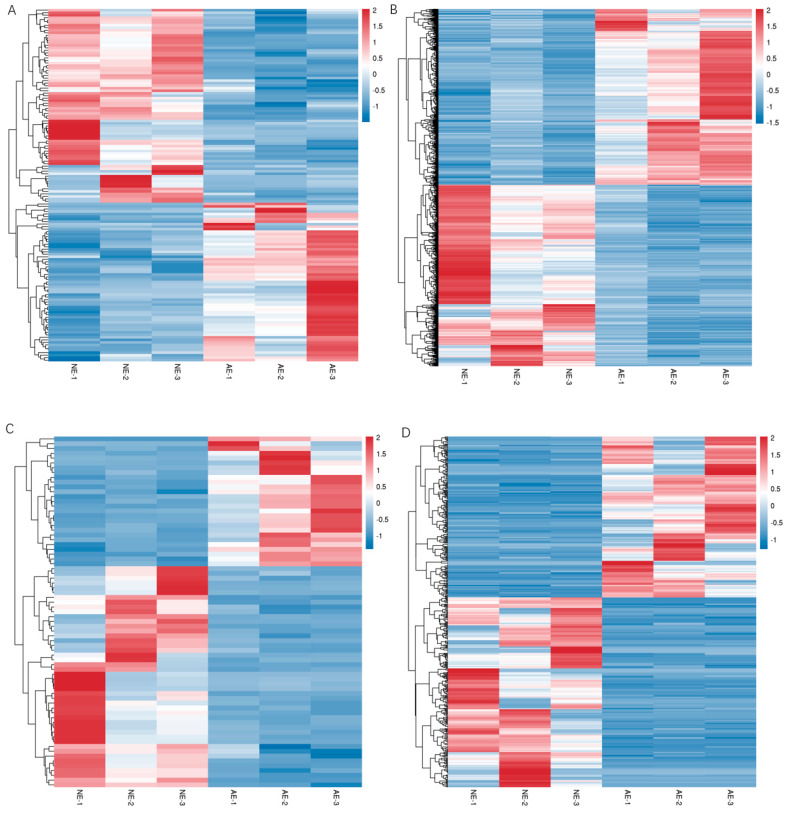
Hierarchical clustering heatmaps of DERNAs in ovarian tissues of NE and AE gilts. (**A**) DEmiRNAs; (**B**) DEmRNAs; (**C**) DElncRNAs; (**D**) DEcircRNAs. Transcripts were screened using the thresholds of FDR < 0.05 and |log_2_FC| ≥ 1.

Hierarchical clustering heatmaps were constructed to visualize the expression patterns of DE RNAs, including miRNAs, mRNAs, lncRNAs, and circRNAs, in ovarian tissues of gilts between the NE and AE groups (Figure 7). Each row represented a DE RNA, and each column represented a sample. The color scale reflected normalized expression levels, with red indicating relatively high expression and blue indicating relatively low expression. DEmiRNAs, DEmRNAs, DElncRNAs, and DEcircRNAs exhibited distinct expression profiles between the two groups, with clear separation of samples from the NE and AE groups, indicating significant transcriptomic differences between the two physiological states under the defined threshold (FDR< 0.05, |log_2_FC| ≥ 1).

### 2.5. Gene Ontology (GO) Enrichment of DEmiRNAs, mRNAs, lncRNAs, and circRNAs in NE/AE Gilt Ovaries

**Figure 8:**
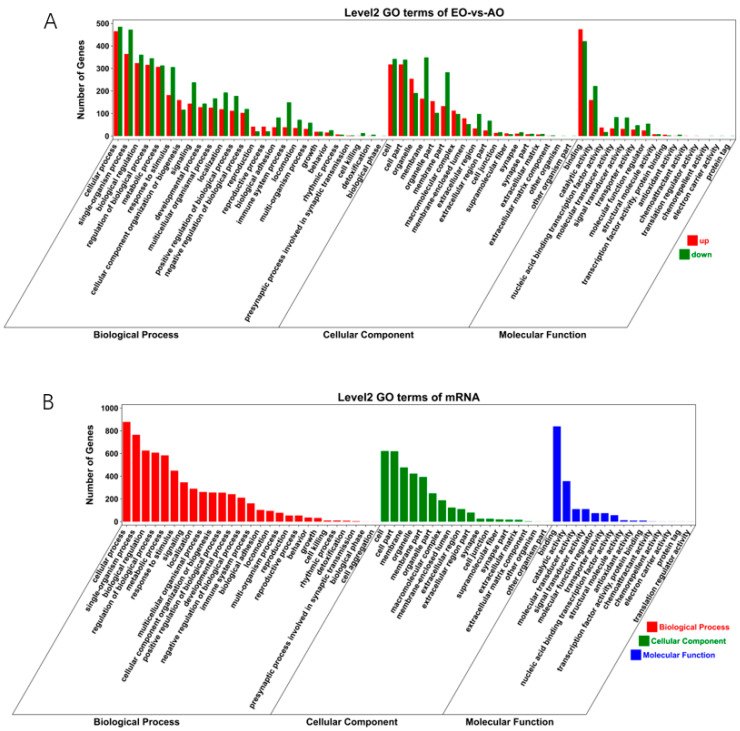

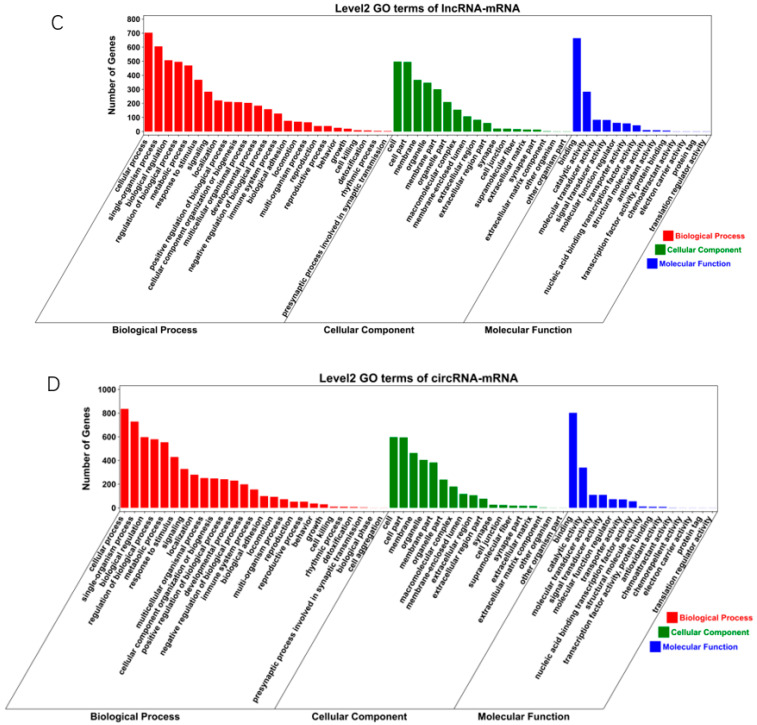
Gene Ontology (GO) enrichment analysis of DERNAs in ovarian tissues of NE and AE gilts. (**A**) DEmiRNAs; (**B**) DEmRNAs; (**C**) DElncRNAs; (**D**) DEcircRNAs.

Gene Ontology (GO) enrichment analysis was performed for the target genes of DEmiRNA, DEmRNA, DElncRNA, and DEcircRNA (Figure 8) to explore the potential functional roles of DE RNAs in the ovarian tissues of gilts during the estrous cycle. The GO terms were classified into three main categories: biological process, cellular component, and molecular function. In the biological process category, growth and development terms such as cellular processes, growth and developmental processes, reproductive processes, negative regulation of biological processes, positive regulation of biological processes, organization or biogenesis of cellular components, metabolic processes, and cell activities aggregation were the most significantly enriched across all RNA types. In the cellular component category, the most abundant terms were cell, cell part, and organelle. For molecular function, binding and catalytic activity were the dominant enriched terms. Through these findings, it was suggested that the DE RNAs may participate in multiple biological processes and molecular functions in ovarian tissues, potentially contributing to the regulation of the estrous cycle.

### 2.6. KEGG Pathway Enrichment of DEmiRNAs, mRNAs, lncRNAs and circRNAs in NE/AE Gilt Ovaries

**Figure 9:**
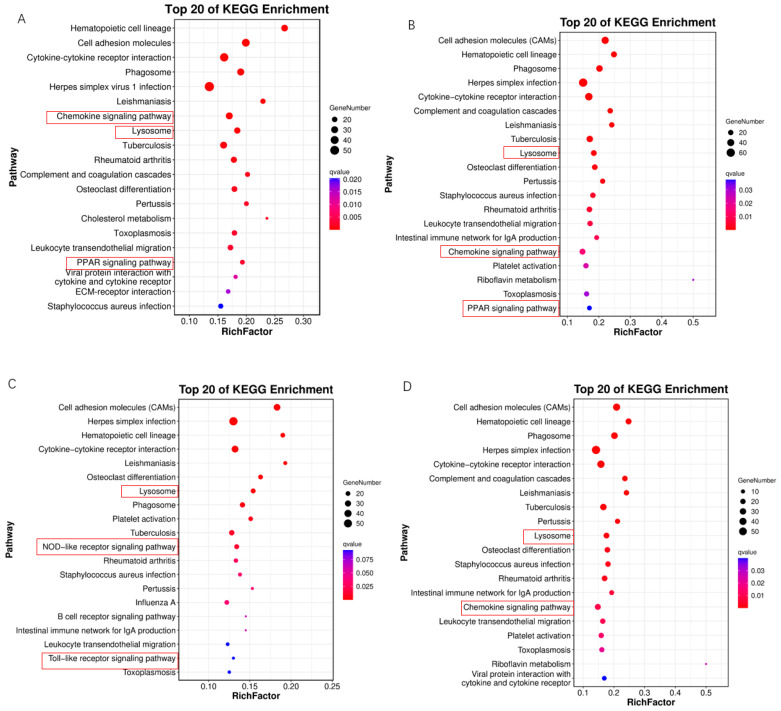
Kyoto Encyclopedia of Genes and Genomes (KEGG) pathway enrichment analysis of DERNAs in ovarian tissues of NE and AE gilts. (**A**) DEmiRNAs; (**B**) DEmRNAs; (**C**) DElncRNAs; (**D**) DEcircRNAs.

KEGG pathway enrichment analysis for the target genes of DEmiRNA, DEmRNA, DElncRNA, and DEcircRNA (Figure 9) was performed to further explore the functional pathways involved in ovarian transcriptomic differences between the NE and AE groups. The top 20 significantly enriched pathways are shown for each RNA type. Commonly enriched pathways across different RNA types included the Chemokine signaling pathway, lysosome, and PPAR signaling pathway, which are closely related to ovarian function, immune regulation, and hormone metabolism. Additionally, specific pathways such as the NOD-like receptor signaling pathway, B cell receptor signaling pathway, and Toll-like receptor signaling pathway were uniquely enriched in certain RNA types. From these results, it was suggested that the differentially expressed RNAs may regulate ovarian physiology and the estrous cycle through multiple signaling pathways, providing important clues for understanding the onset of gilt puberty.

### 2.7. ceRNA Regulatory Network

**Figure 10:**
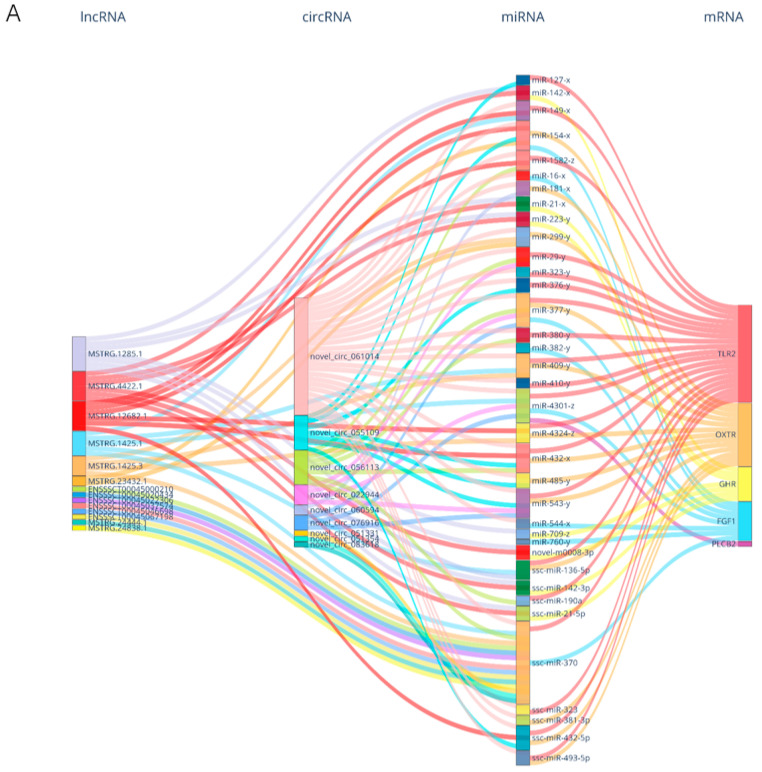

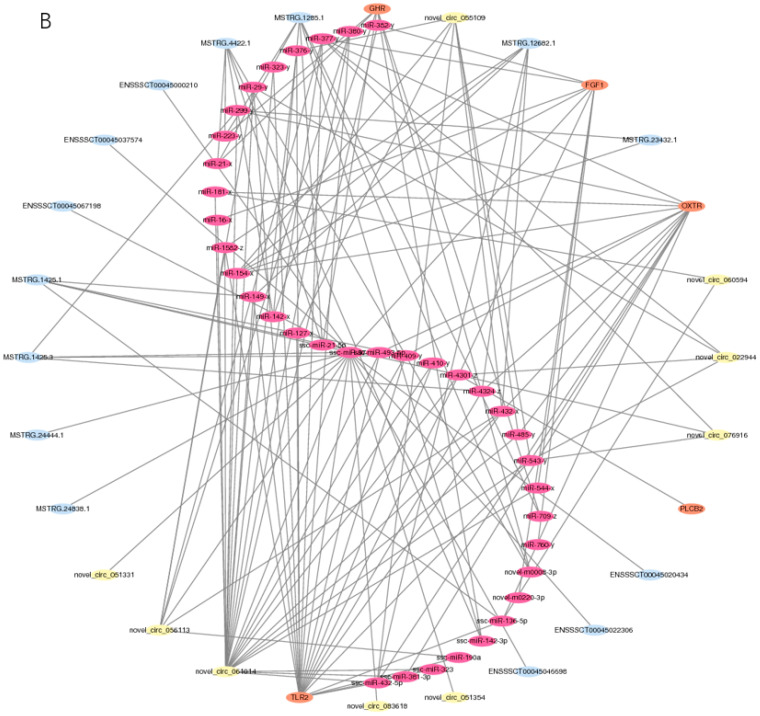
The ceRNA regulatory network in the ovarian transcriptome. (**A**) Sankey diagram showing global interactions among lncRNAs, circRNAs, miRNAs, and mRNAs. (**B**) Node-edge network diagram of the core ceRNA network, where nodes represent different RNA molecules and edges represent regulatory relationships; Node colors represent different RNA types: orange for mRNAs, magenta for miRNAs, light-blue for lncRNAs, and light-yellow for circRNAs.

As shown in Figure 10, the ceRNA regulatory network, consisting of lncRNAs, circRNAs, miRNAs, and mRNAs, was constructed based on miRNA target prediction and expression correlation analysis. All detailed interaction pairs of lncRNA–miRNA–mRNA and circRNA–miRNA–mRNA ceRNA regulatory network are listed in Supplementary Tables Word 1 and Word 2. As shown in the Sankey diagram (Figure 10A) and node-edge network (Figure 10B), multiple lncRNAs (e.g., MSTRG.1285.1, MSTRG.4422.1) and circRNAs (e.g., novel_circ_056113,novel_circ_061014) act as miRNA sponges to competitively bind with miRNAs (e.g., ssc-miR-21-5p, ssc-miR-370), thereby regulating the expression of key ovarian functional genes including *TLR2*, *OXTR*, *GHR*, *FGF1*, and *PLCB2*. Notably, *TLR2* was identified as a core hub mRNA regulated by the largest number of ceRNA pairs, suggesting its potentially critical role in ovarian transcriptomic regulation during estrous cycle disorders.

### 2.8. Quantitative Validation of Estrus-Associated ceRNAs in NE/AE Gilt Ovaries

**Figure 11:**
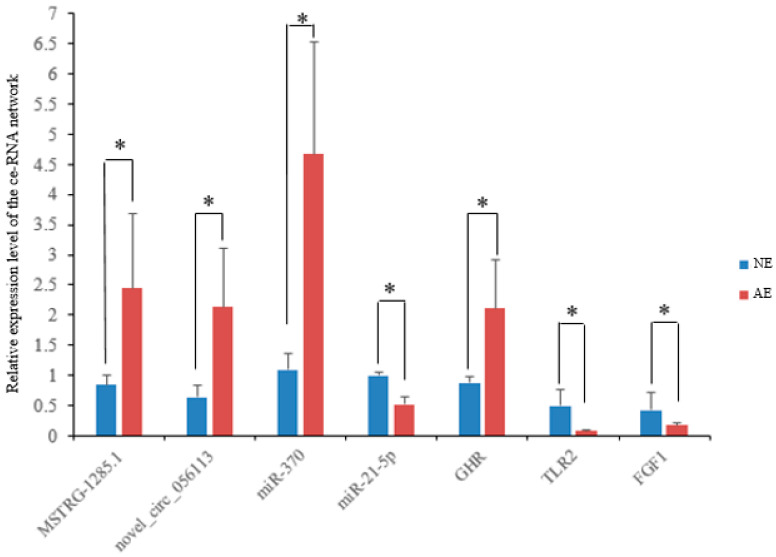
qRT-PCR validation of relative expression levels of lncRNA, circRNA, miRNA, and mRNA in ovarian tissues from NE and AE gilts. Relative expression was calculated via the 2^−ΔΔCt^ method. Data are shown as mean ± SD. Asterisks (*) indicate significant differences between the two groups (*p* < 0.05).

qRT-PCR for seven core ceRNA molecules from the regulatory network, namely one lncRNA (MSTRG.1285.1), one circRNA (novel_circ_056113), two miRNAs (miR-370, miR-21-5p), and three mRNAs (*GHR*, *TLR2*, *FGF1*), was conducted to verify the reliability of RNA-seq data. As illustrated in Figure 11, the expression trends of these transcripts were highly consistent with transcriptome sequencing results. Relative to the NE group, MSTRG-1285.1, novel_circ_056113, miR-370, and *GHR* were significantly upregulated in the AE group. In contrast, miR-21-5p, *TLR2*, and *FGF1* exhibited significantly decreased expression (*p* < 0.05). The differential expression of the candidate ceRNAs was validated through these findings, demonstrating the credibility of our transcriptomic data and suggesting that these RNAs may participate in estrous cycle regulation.

## 3. Discussion

In this study, Landrace × Yorkshire crossbred gilts were selected, and ovarian samples from individuals with two physiological states (normal estrus and follicular cyst-associated anestrus) were sampled for whole-transcriptome sequencing. DERNAs between the two groups were screened, a ceRNA regulatory network was constructed, and the biological functions and signaling pathways enriched by target RNAs were systematically analyzed, aiming to explore key candidate RNAs associated with the estrus of gilts. Hormone levels revealed that NE gilts exhibited significantly higher serum FSH and E_2_ but markedly lower P_4_ concentrations. Ultrasound and anatomical observation demonstrated that, in NE ovaries, uniform and normal follicles are predominant, while in AE ovaries, cystic follicles and luteum appear simultaneously. A comprehensive analysis of serum hormone profiles and ovarian morphological characteristics might indicate that AE gilts are not completely anestrus, and follicular cysts are the key factor inducing silent estrus [21,22]. These findings provide morphological and endocrine evidence for interpreting estrous disorders in gilts. A total of twelve sequencing libraries were constructed in this research. The classification of RNA, sequence length, number of exons, and expression levels all conform to the typical sequence characteristics of various RNAs, verifying the high quality of sequencing data and laying a solid foundation for subsequent screening of DERNAs and construction of the ceRNA regulatory network. The PCA results showed that the sequencing data had reliable repeatability, and the overall expression characteristics of the ovarian transcriptomes in the NE and AE gilts were significantly separated. A large number of DERNAs were identified under the preset screening thresholds. Volcano plots and hierarchical clustering heatmaps collectively revealed extensive transcriptional discrepancies in ovaries under the two physiological states, supplying abundant candidate RNAs for subsequent ceRNA interaction analysis.

To characterize transcriptomic signatures underlying this silent estrus phenotype triggered by follicular cysts, we performed GO functional enrichment analysis on target genes of differentially expressed miRNAs, mRNAs, lncRNAs, and circRNAs. The results illustrated that these target genes were predominantly enriched in biological categories tightly linked to gilt ovarian reproductive endocrine activity, including cellular biological processes, growth and development, reproductive regulation, and substance metabolism. Nevertheless, the top enriched GO terms such as binding, cellular process, and metabolic process are overly broad and lack tissue-specific information for interpreting estrous cycle disorders. Meanwhile, using KEGG pathway enrichment analysis, seven significantly enriched signaling pathways, namely lysosome pathway, PPAR signaling pathway, chemokine signaling pathway, cholesterol metabolism pathway, NOD-like receptor (NLR) signaling pathway, B cell receptor signaling pathway, and Toll-like receptor (TLR) signaling pathway, were uncovered. Consistent with our results, Takenaka et al. [23] previously verified that the lysosome pathway is tightly involved in mammalian reproductive modulation. Hydrolases enriched in lysosomes can degrade ovarian surface epithelial cells to promote ovulation; in addition, this pathway also participates in the synthesis and secretion of steroid hormones that dominate reproductive activities. In another study focusing on dairy cow estrus, Liu et al. [24] identified an activated cholesterol metabolism pathway via miRNA enrichment analysis of milk exosomes, with the close correlation between cholesterol metabolism and animal estrus regulation further confirmed. TLR and NLR represent two core families of pattern recognition receptors (PRRs) that exert critical functions in host innate immunity, both of which can be triggered by endogenous and exogenous danger signals. Cumulative evidence has proven extensive crosstalk between TLR and NLR signaling pathways. For example, Liu et al. [25] reported that estrogen insufficiency upregulates *TLR2* and *TLR4* expression, which further activates the hippocampal NLRP inflammasome, with the secretion of pro-inflammatory cytokines including IL-1β and IL-18 enhanced. In addition, bidirectional crosstalk exists between the neuroendocrine system and immune system under immune stress. The activation of TLR4 signaling can trigger a downstream NF-κB signaling cascade and inducing the massive release of inflammatory cytokines, which in turn, exerts pivotal regulatory effects on neuroendocrine homeostasis [26]. Collectively, TLR and NLR signaling pathways are speculated to modulate gilt neuroendocrine balance, further affecting estrus onset. Accordingly, the enriched KEGG pathways screened in our study are strongly correlated with the estrus regulation mechanism of gilts.

Considering that both lncRNAs and circRNAs function as miRNA sponges to competitively bind and inhibit miRNAs, an lncRNA/circRNA–miRNA–mRNA competing endogenous RNA network related to gilt estrus was further constructed in this study. This network helped to reveal the underlying regulatory mechanism whereby lncRNAs and circRNAs modulate downstream mRNAs, with the target genes synergistically regulated by these two non-coding RNA subtypes identified.

lncRNAs are defined as non-protein-coding transcripts with a length of more than 200 nucleotides [27]. Accumulating evidence has demonstrated that lncRNAs participate in multiple ovarian reproductive processes, including ovarian follicular development, granulosa cell proliferation, and hormone secretion [28]. Previous porcine studies have also found that numerous lncRNAs are markedly downregulated during oocyte maturation, and their decreased expression facilitates oocyte meiotic maturation, with subsequent developmental competence improved [29,30]. In line with these previous findings, our results revealed that lncRNA MSTRG-1285.1 exhibited low expression levels in ovarian tissues of NE gilts. We speculated that MSTRG-1285.1 serves as a pivotal regulatory molecule: its reduced expression modulates meiotic progression, cell cycle, and cytoplasmic maturation pathways, with porcine oocyte maturation during meiosis accelerated and the onset of estrus in gilts ultimately promoted.

circRNAs are a unique category of circular non-coding RNAs, which exert vital regulatory effects on the growth and development of primordial follicles, ovaries, and uteruses in mammals [28,29,30,31]. As typical miRNA molecular sponges, circRNAs participate in post-transcriptional gene regulation during the estrous cycle of Xiang pigs [32]. Previous transcriptomic research on pubertal gilts has characterized the expression profiles of ovarian circRNAs. Functional enrichment analysis further demonstrated that the host genes of these circRNAs are primarily involved in progesterone-mediated oocyte maturation and steroid biosynthesis pathways, both of which are closely linked to reproductive processes [33]. In line with these previous findings, the present study identified significant downregulation of novel_circ_056113 in the ovarian tissues of NE gilts. A potential regulatory mechanism underlying this circRNA was proposed: decreased expression of novel_circ_056113 diminishes its competitive sponging activity against target miRNAs. These released miRNAs suppress downstream estrus-inhibitory genes and activate estrus-stimulatory genes. This process initiates estrus-related signaling pathways and eventually drives the onset and progression of the estrous cycle in gilts.

Target gene prediction for the identified differentially expressed miRNAs revealed that the highly expressed ssc-miR-370 binds to *FGF1* and *TLR2* in gilt ovaries throughout various estrous stages. By contrast, GHR was predicted as a target of ssc-miR-21-5p. Meanwhile, KEGG enrichment analysis of the target genes of these miRNAs uncovered multiple pathways associated with reproduction, such as the lysosome pathway, PPAR signaling pathway, chemokine signaling pathway, and cholesterol metabolism pathway. Accumulating evidence demonstrates that miR-370 plays a key regulatory role in ovarian follicle development [32]. Zhao et al. [34] previously reported that ssc-miR-370 upregulation correlates positively with the estrus rate of gilts. Its high expression also remodels the miRNA landscape in the endometrium, which facilitates reproductive activity and pregnancy establishment.

In another transcriptomic analysis of pigs, Li et al. [35] applied high-throughput sequencing to compare endometrial miRNA profiles between pregnant and non-pregnant Meishan and Yorkshire pigs. Their data confirmed that ssc-miR-21 was the most highly expressed miRNA in both breeds, suggesting that this molecule is essential for embryo implantation in pigs. Using high-throughput sequencing, Liu et al. [36] explored the effects of acupuncture on rat ovaries. The results revealed that acupuncture could alleviate poor ovarian response (POR) in rats via modulating miR-154-5p. The therapeutic effect of acupuncture on POR is probably associated with the downregulation of miR-154-5p and suppressed cellular apoptosis. Beyond the two key miRNAs involved in our ceRNA network, we also detected obvious upregulation of miR-485-y in ovarian tissues of anestrous gilts. Existing studies have demonstrated that miR-485-y regulates ovarian hormone metabolism and granulosa cell proliferation, thus facilitating estrous cycle progression [36,37]. Mechanistically, miR-485-y targets the oxytocin receptor gene (OXTR), a critical factor triggering estrus in gilts [38]. Oxytocin (OT) binds to receptors on the endometrial cell membrane and activates downstream signaling cascades, which in turn boosts the secretion of prostaglandin F2α (PGF2α). Secreted PGF2α acts on ovarian luteal cells to stimulate OT release, creating a positive feedback loop that triggers luteolysis—a key physiological event marking the initiation of estrus [39]. Additionally, Singh et al. [40] documented dynamic expression changes of salivary miR-16 across the estrous cycle. Its expression was markedly higher on day 6 and days 18–19 than on estrus day (day 0), day 10, and the subsequent estrus phase. Such expression patterns may result from the upregulation of its target genes (including *FGF2*, *BDNF*, *KRAS*, and IGF1) in granulosa cells of antral follicles. These genes play dominant roles in follicle development and estrus regulation around the estrous period.

Five hub genes retrieved from the ceRNA regulatory network in the present study have all been demonstrated to regulate the estrous cycle in animals. Grazul-Bilska et al. [41] demonstrated that fibroblast growth factors *FGF1* and *FGF2* promote luteal cell proliferation and progesterone secretion in sheep during the luteal phase. Luteal cells isolated from the early luteal phase exhibit the strongest proliferative and progesterone-synthetic responses to *FGF1* treatment. Overall, FGF family members act as key modulators of luteal cell growth and differentiation in ewes [41]. Schutz et al. [42] further verified the regulatory role of FGF signaling in bovine follicular development. FGFs and their receptors inhibit ovarian cell differentiation, and steroid hormones modulate the expression of FGF and FGFR, which in turn governs the selection of dominant follicles. These results collectively suggest that FGF signaling is essential for normal estrus regulation in livestock. OXTR, another core reproductive gene identified in the network, is highly expressed in the ventromedial hypothalamus (VMH) and participates in the control of female sexual behavior [43]. Estrogen elevates OXTR expression in the VMH, thereby amplifying oxytocin-related behaviors. This mechanism is closely associated with locomotor activity in female rats at the proestrus stage [43]. In line with these observations, Wijesena et al. [38] reported that gilts with delayed puberty presented reduced ovarian expression of folliculogenesis-associated genes including OXTR. This downregulation stems from inadequate gonadotropin release and diminished ovarian responsiveness, ultimately postponing estrus initiation. In addition, Kanthawat et al. [44] observed that ovarian OXTR protein levels reach a maximum in the follicular phase and decline markedly after conception. This distinct expression pattern implies that OXTR facilitates cervical relaxation and sperm transport to support normal estrus and mating. During pregnancy, by contrast, it helps maintain cervical constriction to safeguard fetal development.

Growth hormone receptor (GHR) exerts the biological effects of growth hormone (GH) through specific ligand-receptor interactions [45]. Accumulating studies have shown that GHR expression fluctuates periodically during the mammalian estrous cycle. In dairy cows, GHR abundance rises in the uterus and ovaries at the estrous stage [46]. Long-term analysis of endometrial samples from cows across distinct cycle phases further verified the stage-dependent expression pattern of GHR [47]. Rhoads et al. [46] examined GHR levels in the liver and endometrium of dairy cows at four lactation stages. Their results revealed that uterine GHR mRNA expression peaked during estrus, indicating that increased GHR expression is tightly linked to normal estrus in cattle. In agreement with the above reports, this study also detected markedly higher GHR expression in ovarian tissues of gilts with regular estrus.

*TLR2* displays expression patterns that fluctuate alongside the reproductive cycle in the female reproductive tract. In sheep, uterine *TLR2* mRNA levels change markedly according to physiological status, showing temporal and cell-type-specific expression in the endometrium during the estrous cycle and early gestation [48]. Mechanistically, *TLR2* triggers the downstream NF-κB signaling pathway and elevates the expression of pro-inflammatory cytokine IL-6, thereby repressing the transcription of ovulation-associated genes [49]. It is, therefore, hypothesized that *TLR2* high expression inhibits estrus, whereas its low expression facilitates normal estrous cycle progression. In the present study, ovarian *TLR2* expression was significantly lower in NE gilts than in AE counterparts, which aligns with the above regulatory mechanism. Additionally, Hickey et al. [50] reported sustained *TLR2* expression in the uterus and vagina of mice across the entire estrous cycle, with its abundance varying at different stages. This finding further evidences that *TLR2* acts as a key regulator of estrous cycles in animals.

Phospholipase C is involved in the secretion of estradiol and progesterone, and thus, modulates estrus in pigs [51]. As a major isoform of phospholipase C, *PLCB3* serves as a central regulator of ovarian ovulation, luteinization, and follicular development [52,53]. Studies on cattle have shown that luteinizing hormone (LH) regulates ovarian physiology through luteinizing hormone receptor (LHR) signaling, and *PLCB3* is essential for granulosa cell differentiation [54]. Knockdown of *PLCB3* weakens LH-induced granulosa cell differentiation, lowers aromatase levels, and suppresses estradiol production [52]. Given the generally conserved biological functions of homologous genes across livestock species, we tentatively hypothesize that the differentially expressed *PLCB2* identified in gilt ovaries may exert partially comparable reproductive effects on porcine ovulation, corpus luteum formation, and follicular growth; however, this extrapolation remains a cautious assumption, as *PLCB2* and *PLCB3* are distinct PLC isoforms with no direct experimental evidence confirming their functional interchangeability in pig ovaries. Supporting this tentative hypothesis indirectly, transcriptomic analysis of goat hypothalamic tissues indicated that *PLCB2* interacts with numerous lncRNAs to control the secretion of major reproductive hormones, such as GnRH, oxytocin, and E2 [55].

qRT-PCR analysis was performed on seven hub RNAs to verify the reliability of our transcriptomic sequencing data and construct a ceRNA regulatory network. Consistent with the RNA-seq results, MSTRG-1285.1, novel_circ_056113, miR-370, and *GHR* were significantly upregulated in AE gilts with ovarian dysfunction. In contrast, miR-21-5p, *TLR2*, and *FGF1* exhibited marked downregulation. The expression patterns of these key molecules were consistent with previously reported transcriptomic profiles of porcine ovarian cystic lesions. Functional analysis indicated that elevated *GHR* expression may facilitate aberrant granulosa cell proliferation in cystic ovaries. Meanwhile, the reduced abundance of miR-21-5p and *FGF1* impairs the signaling cascades governing normal follicular development, thereby inducing follicular atresia and ovarian cyst formation. Collectively, this study identifies novel biomarker candidates and core regulatory pathways, providing valuable insights into the molecular mechanisms underlying ovarian cyst pathogenesis in gilts.

There are several study limitations to the present study that should be noted.

(1) Whole-transcriptome sequencing was conducted with merely three biological replicates per group. While n = 3 is the widely accepted empirical minimum for DESeq2 analysis, this small sample size weakens statistical power and limits the generalizability of transcriptomic results; inter-individual hormonal divergence among gilts may further obscure transcripts with mild differential expression signals.
(2) Reproductive hormones (FSH, E2, and P4) fluctuate substantially throughout the porcine estrous cycle, serving as a critical confounding factor when comparing endocrine traits between NE and AE gilts. Although we uniformly collected NE gilt samples on estrous cycle day 16 (the stage of luteal regression and early follicular growth) to minimize phase-related bias, minor individual variation in luteolysis and follicle emergence may still alter circulating hormone levels and confound intergroup comparisons.
(3) We could not perform luteal marker IHC to validate functional corpora lutea. As ovarian tissues were used for macroscopic morphological observation, constructed sequencing libraries, and consumed for RNA extraction, no spare tissue material was retained.
(4) The ceRNA sponge relationship of MSTRG.1285.1 and novel_circ_056113 with miR-370/miR-21-5p was only supported by target prediction and qRT-PCR correlation. Dual-luciferase, knockdown, and overexpression assays in granulosa cells are needed to verify their direct binding, which were not conducted here.

## 4. Materials and Methods

### 4.1. Sample Collection

Experimental animals were 180-day-old Landrace × Yorkshire crossbred gilts with similar body weight and health status, all of which were half-siblings. All pigs were reared under identical housing and management conditions in the same pen. Estrus detection was conducted twice daily (morning and evening) using a mature boar. Gilts displaying typical estrus symptoms were subjected to ovarian ultrasonography with a HONDA ELECTRONICS HS-1600V (HONDA ELECTRONICS CO., LTD., Toyohashi, Japan) scanner fitted with an HCS-736M transducer. The device was set to an abdominal imaging mode at a frequency of 5.0 MHz, with MI, TI, and other parameters calibrated to optimize image quality. After applying acoustic coupling gel to the probe, we scanned the lower abdomen to capture ultrasonic images of bilateral ovaries. Combined with morphological observation after slaughter, these gilts were classified as having normal first estrus onset.

NE gilts were sampled on day 16 of the estrous cycle, when corpora lutea begin to regress, and ovarian follicles start to develop. A threshold of 230 days was adopted to screen AE gilts, in accordance with local commercial gilt breeding standards and previous porcine reproductive studies [56]. Gilts that showed no estrous signs after sustained boar stimulation until 230 days were subjected to ovarian ultrasonography following the same protocol, and post-slaughter morphological examination verified that these gilts failed to initiate their first estrus and thus were diagnosed as anestrous individuals. Post-slaughter ovarian tissues were collected from NE and AE gilts.

Finally, three gilts with normal first estrus and three with anestrus were selected. The whole intact ovarian tissues were harvested immediately after slaughter, rinsed with normal saline, snap-frozen in liquid nitrogen, and then preserved at −80 °C for subsequent experiments.

### 4.2. Measurement of Serum FSH, E2 and P4 Levels in NE/AE Gilts

Blood samples (10 mL) were collected from the jugular vein of NE and AE gilts. Samples were centrifuged at 3500 rpm for 15 min, and the resulting serum supernatants were harvested and stored at −80 °C until use. Serum concentrations of FSH, E_2_, and P_4_ were quantified using commercial ELISA kits (Beijing Keruimei Technology Co., Ltd., Beijing, China). For the Pig FSH ELISA Kit, the sensitivity was 0.78 mIU/mL, with intra-assay CV < 8% and inter-assay CV < 10%. For the Pig E_2_ ELISA Kit, the sensitivity was 14.45 pg/mL, with intra-assay CV < 8% and inter-assay CV < 10%. For the Pig P_4_ ELISA Kit, the sensitivity was 0.49 ng/mL, with intra-assay CV < 8% and inter-assay CV < 10%.

### 4.3. Total RNA Extraction, Library Construction and Sequencing

Total RNA was isolated from fully homogenized intact whole ovaries using TRIzol reagent, and no isolated follicular tissue was used for RNA extraction (No separate follicular tissue was dissected individually, and no enrichment of any specific ovarian structures was performed throughout this experiment). RNA concentration and purity were evaluated with a NanoDrop spectrophotometer. After reverse transcription into complementary DNA (cDNA), strand-specific RNA libraries were constructed, and whole transcriptome sequencing was performed on the Illumina HiSeq 2500 (Illumina, Inc., San Diego, CA, USA) platform. All sequencing services were provided by Guangzhou Genedenovo Biotechnology Co., Ltd. (Guangzhou, China).

### 4.4. Analysis of Transcriptome Sequencing Data

Raw sequencing data were evaluated and filtered using FastQC and NGSQC Toolkit to trim adapter sequences and low-quality reads, yielding clean reads. The qualified reads were then mapped to the pig reference genome Sus scrofa 11.1 using TopHat2. Transcript assembly was performed with Cufflinks and Cuffmerge. The coding potential of mRNAs, lncRNAs, and circRNAs was predicted via FeatureCounts, CNCI, CPC, and CIRI2. In addition, miRDeep2 was used for miRNA identification.

Normalized expression data of the four RNA types were subjected to principal component analysis (PCA) in the R package, version 2.8. Sample distribution was visualized based on the first two principal components (PC1 and PC2), with the corresponding variance contributions annotated on axes. The 95% confidence ellipses were applied to illustrate the similarity among biological replicates within each group.

### 4.5. Differential Expression Analysis of miRNA, mRNA, lncRNA, and circRNA

Differential expression analysis of miRNAs, mRNAs, lncRNAs, and circRNAs was conducted with the DESeq2 R package. Transcripts were identified as differentially expressed according to the screening thresholds below: mRNAs and lncRNAs were filtered at FDR < 0.05 and |log_2_FC| ≥ 1, whereas miRNAs and circRNAs were filtered at *p* < 0.05 and |log_2_FC| ≥ 1.

### 4.6. Prediction and Functional Enrichment Analysis of Target Genes for DERNAs

(1) Target genes of miRNAs were predicted using TargetScan and miRanda. Gene Ontology (GO) annotation was conducted via the DAVID online tool (https://david.ncifcrf.gov/conversion.jsp (accessed on 10 May 2025), and Kyoto Encyclopedia of Genes and Genomes (KEGG) enrichment analysis was performed using WebGestalt (http://www.webgestalt.org/ (accessed on 10 May 2025). Enrichment results with *p* < 0.05 were regarded as statistically significant. All visualizations were completed with the R package ggplot2.
(2) TRRUST and JASPAR were applied to predict target genes of mRNAs. GO and KEGG enrichment analyses were carried out using the R package clusterProfiler, and gene set enrichment analysis (GSEA) was implemented accordingly. The resulting data were visualized using ggplot2.
(3) Bedtools was used to screen cis-target genes located within 50 kb upstream and downstream of differentially expressed lncRNAs. Spearman’s correlation analysis between expression profiles of lncRNAs and mRNAs was performed to predict their trans-target genes.
(4) CircRNAs were identified/filtered with Findcirc. All detected circRNAs were reordered by length from shortest to longest and renamed. circAnno was used to annotate their host transcripts and parent genes, followed by sequence alignment against the circBase database to label known circRNAs. Expression quantification was performed using the transcripts per million (TPM) normalization method.

### 4.7. ceRNA Regulatory Network Construction and Analysis

RNAhybrid was used to predict the targeting interactions between lncRNAs, circRNAs, and miRNAs according to sequence complementarity. Combined with their target gene profiles, Pearson correlation analysis and Cytoscape (version 3.9.1) were applied to construct/visualize lncRNA-miRNA-mRNA and circRNA-miRNA-mRNA ceRNA regulatory networks.

### 4.8. Reverse Transcription Quantitative PCR (RT-qPCR) Validation of the Estrus-Related RNAs

One lncRNA (MSTRG.1285.1), one circRNA (novel_circ_056113), two miRNAs (miR-370 and miR-21-5p), and three mRNAs (GHR, TLR2, and FGF1) were selected for expression validation via quantitative real-time PCR (qRT-PCR). Total RNA integrity was assessed via agarose gel electrophoresis, while RNA purity and concentration were determined using a NanoDrop 2000 spectrophotometer (Thermo Fisher Scientific, Wilmington, USA). Only RNA samples with OD_260_/OD_280_ between 1.8 and 2.1 and OD_260_/OD_230_ > 1.8 were retained for subsequent reverse transcription. For mRNA, lncRNA, and circRNA: 1 μg of qualified total RNA was reverse-transcribed into complementary DNA (cDNA) using a commercial reverse transcription kit following the manufacturer’s protocols. For miRNA: Specific stem-loop reverse transcription primers were used to synthesize miRNA cDNA from equal amounts of total RNA. Primers were designed with DNAStar 6.1.3, and all sequences were presented in Table 1. Primers were synthesized by Sangon Biotech (Shanghai) Co., Ltd. qPCR amplification was performed on a LightCycler 96 Real-Time PCR System. The total reaction volume was 10 μL, consisting of 5 μL SYBR Green qPCR Master Mix, 1 μL of each forward and reverse primer (2 μmol/L), 2 μL of cDNA template, and 1 μL of RNase-free double-distilled water. The thermal cycling program was set as follows: initial denaturation at 95 °C for 3 min; followed by 40 cycles of denaturation at 95 °C for 10 s, annealing at 60 °C for 30 s, and extension at 72 °C for 20 s. After amplification, a melting curve analysis ranging from 65 °C to 95 °C with a temperature increment of 0.5 °C per step was performed to confirm the specificity of each amplified product. Single sharp melting peaks indicated no non-specific amplification or primer dimer formation. The relative expression levels of target transcripts were calculated using the 2^−ΔΔCt^ method. Specifically, β-Actin and GAPDH served as dual reference genes for normalization of mRNAs, lncRNAs and circRNAs, whereas U6 snRNA was used as the internal control for miRNAs. Three independent biological replicates were used for each experimental group, and each replicate consisted of ovarian tissue collected from an individual gilt; no pooled samples were adopted.

### 4.9. Statistical Analysis of Experimental Data

Raw data were collated and sorted in Microsoft Excel. Statistical analyses were performed using SPSS 26.0 software. Differences in serum reproductive hormone levels between the two groups (NE gilts vs. AE gilts) were assessed via an independent samples *t*-test. Three biological replicates were included for each group. All data are expressed as mean ± standard deviation (SD). Statistical significance was defined as *p* ≤ 0.01 (marked with **) and *p* < 0.0001 (marked with ****).

## 5. Conclusions

In summary, we systematically compared the structural features and expression profiles of miRNAs, mRNAs, lncRNAs, and circRNAs in ovarian tissues from NE and AE gilts. Our transcriptomic data indicated abundant expression of non-coding RNAs (ncRNAs) in ovarian samples. Subsequent bioinformatic enrichment analyses suggested that these differentially expressed ncRNAs may participate in multiple biological cascades, including lysosome function, the PPAR signaling pathway, the chemokine signaling pathway, and the NOD-like receptor signaling pathway. Based on target prediction and expression correlation, these ncRNAs are hypothesized to function as candidate competing endogenous RNAs (ceRNAs) that might modulate pivotal ovarian transcripts (e.g., FGF1, GHR, TLR2) by interacting with ssc-miR-370, miR-21-5p, and other functional miRNAs. Integrating post-transcriptional regulation and neuroendocrinology, this work provides fundamental data and valuable references for further exploring gilt reproductive performance at the transcriptomic level.

## Figures and Tables

**Table 1:** List of lncRNA/circRNA-miRNA-mRNA and their primer sequences.

Name	Primer Type	Sequence (5′–3′)	Amplicon Size (bp)
β-Actin	F	TGGTTCCTGGGCTCTGTAAGGC	60
	R	TGATGCCGTGTTCTATTGGGTA	
MSTRG.1285.1	F	TTCTTTCCTCTCCAAGCCCAG	266
	R	AAGAAAAGGGGCTGTCGTGGA	
novel_circ_056113	F	TTCCCTTTGAGAGTGGACCTGATATT	135
	R	TGGAAGAGAGATGTCCCTGTTTATGA	
U6	F	CTCGCTTCGGCAGCACA	94
	R	AACGCTTCACGAATTTGCGT	
miR-21-5p	F	CGTAGCTTATCAGACTGATGTTGAA	79
ssc-miR-370	F	ACACTCCAGCTGGGGCCGCTGGGGGTGGAA	66
Universal miRNA R	UR-PloyA	GCTGTCAACGATACGCTACGTAAC	—
GAPDH	F	GACATCAAGAAGGTGGTGAAGCA	177
	R	GTCGTACCAGGAAATGAGCTTGA	
FGF1	F	CACCCAGTGAGGAGTGTTTTGTTC	212
	R	GTCAGCACCCCAGGTCATTTCTT	
GHR	F	AAGAAATGGAAAAGAGTGCCCTGAT	195
	R	TCAGTAGAGTCCAGTTGAGGCCA	
TLR2	F	AAAGCCTTGACCTGTCCAATAAC	117
	R	CTTCCTCCACGGTATGAATGCTA	

Note: For miRNA quantitative detection, universal reverse primer UR-PloyA was shared by miR-21-5p and ssc-miR-370; F = forward primer, R = reverse primer; Amplicon size indicates predicted length of PCR product.

## Data Availability

The original contributions presented in this study are included in the article. Further inquiries can be directed to the corresponding author.

## Supplementary Materials

The following supporting information can be downloaded at: https://www.mdpi.com/article/10.3390/ijms27156848/s1. The raw sequence data reported in this paper have been deposited in the Genome Sequence Archive, China National Center for Bioinformation/Beijing Institute of Genomics, Chinese Academy of Sciences (GSA: CRA031534) that are publicly accessible at https://ngdc.cncb.ac.cn/gsa, accessed on 7 June 2026.
