# Supplementary material for: Whole-Transcriptome Profiling of Ovarian Tissues in Gilts with Normal Estrus and Follicular Cyst-Associated Anestrus
Source: Int J Mol Sci. 2026 Jul 30;27(15):6848. doi: 10.3390/ijms27156848 (PMC13467522; doi:10.3390/ijms27156848)
Supplement: Supplementary file 1 [file ijms-27-06848-s001.zip › mRNA-lncRNA-circRNA Total RNA library depleted of rRNA-supplementary tables.pdf]

Table S7. Data filtering statistics table of ovraies in gilts

Sample	RawDatas	CleanData(%)	Adapter(%)	LowQuality(%)	polyA(%)	N(%)
EO-1	89880446	89581346 (99.67%)	70258 (0.08%)	228362 (0.25%)	0 (0.00%)	480 (0.00%)
EO-2	77542132	77218020 (99.58%)	49782 (0.06%)	274116 (0.35%)	0 (0.00%)	214 (0.00%)
EO-3	89597002	89323092 (99.69%)	58308 (0.07%)	215574 (0.24%)	0 (0.00%)	28 (0.00%)
AO-1	80568362	80299220 (99.67%)	58782 (0.07%)	209568 (0.26%)	0 (0.00%)	792 (0.00%)
AO-2	88242218	87990524 (99.71%)	47248 (0.05%)	204386 (0.23%)	0 (0.00%)	60 (0.00%)
AO-3	82213570	81859694 (99.57%)	51604 (0.06%)	302272 (0.37%)	0 (0.00%)	0 (0.00%)

Note: EO(Estrus Ovray); AO(Abnormal Ovray), The following table is the same.

Table S8. Base information statistics table of ovraies in gilts

Sample	RawData (bp)	BF_Q20(%)	BF_Q30(%)	BF_N(%)	BF_GC(%)	CleanData (bp)	AF_Q20(%)	AF_Q30(%)	AF_N(%)	AF_GC(%)
EO-1	13482066900	13194919194 (97.87%)	12669735235 (93.97%)	168163 (0.00%)	6937629803 (51.46%)	1337101671	13102667961 (98.00%)	12586657273 (94.14%)	160622 (0.00%)	6875471088 (51.42%)
EO-2	11631319800	11261293597 (96.82%)	1067124585 (91.75%)	110453 (0.00%)	5896930412 (50.70%)	11543094194	11192639128 (96.96%)	10611468887 (91.93%)	106576 (0.00%)	5848785054 (50.67%)
EO-3	13439550300	13111742726 (97.56%)	12554934456 (93.42%)	68088 (0.00%)	6953334374 (51.74%)	13347999592	13038543250 (97.68%)	12489826542 (93.57%)	67292 (0.00%)	6901875847 (51.71%)
AO-1	12085254300	11757304360 (97.29%)	11194520209 (92.63%)	136218 (0.00%)	6016240849 (49.78%)	12005988761	11694506259 (97.41%)	11138885380 (92.78%)	123211 (0.00%)	5972342151 (49.74%)
AO-2	13236332700	12924487721 (97.64%)	12389333610 (93.60%)	68008 (0.00%)	6492363478 (49.05%)	13154621505	12859039406 (97.76%)	12331077159 (93.74%)	66882 (0.00%)	6447379053 (49.01%)
AO-3	12332035500	11957582573 (96.96%)	11347789889 (92.02%)	29222 (0.00%)	5985499276 (48.54%)	12230866313	11877067075 (97.11%)	11276840028 (92.20%)	28977 (0.00%)	5928167612 (48.47%)

Table S9. Alignment results of clean reads to the Ribosome RNA (rRNA) of ovraies in gilts

Sample	Clean_reads	Mapped_Reads(%)	Unmapped_Reads(%)
EO-1	89581346	559972 (0.63%)	89021374 (99.37%)
EO-2	77218020	324466 (0.42%)	76893554 (99.58%)
EO-3	89323092	459976 (0.51%)	88863116 (99.49%)
AO-1	80299220	337658 (0.42%)	79961562 (99.58%)
AO-2	87990524	644722 (0.73%)	87345802 (99.27%)
AO-3	81859694	3575738 (4.37%)	78283956 (95.63%)

Table S10. Alignment results of rRNA-depleted clean reads to the reference genome of ovraies in gilts

Sample	Total	Unmapped(%)	Unique_Mapped(%)	Multiple_Mapped(%)	Total_Mapped(%)
EO-1	89021374	8181494 (9.19%)	78161226 (87.80%)	2678654 (3.01%)	80839880 (90.81%)
EO-2	76893554	7621336 (9.91%)	66807005 (86.88%)	2465213 (3.21%)	69272218 (90.09%)
EO-3	88863116	8154957 (9.18%)	78053108 (87.84%)	2655051 (2.99%)	80708159 (90.82%)
AO-1	79961562	7422955 (9.28%)	70505953 (88.17%)	2032654 (2.54%)	72538607 (90.72%)
AO-2	87345802	7528653 (8.62%)	77293051 (88.49%)	2524098 (2.89%)	79817149 (91.38%)
AO-3	78283956	10079652 (12.88%)	66518212 (84.97%)	1686092 (2.15%)	68204304 (87.12%)

Table S11. Comparison of statistical conditions in the reference area of ovraies in gilts

Sample	Exon	Intron	Intergenic
EO-1	58098068 (71.87%)	20568008 (25.44%)	2173804 (2.69%)
EO-2	49741954 (71.81%)	17735406 (25.60%)	1794858 (2.59%)
EO-3	59470808 (73.69%)	19275671 (23.88%)	1961680 (2.43%)
AO-1	49127251 (67.73%)	20974246 (28.91%)	2437110 (3.36%)
AO-2	55427703 (69.44%)	21891045 (27.43%)	2498401 (3.13%)
AO-3	44973640 (65.94%)	20736541 (30.40%)	2494123 (3.66%)
