# Supplementary material for: Whole-Transcriptome Profiling of Ovarian Tissues in Gilts with Normal Estrus and Follicular Cyst-Associated Anestrus
Source: Int J Mol Sci. 2026 Jul 30;27(15):6848. doi: 10.3390/ijms27156848 (PMC13467522; doi:10.3390/ijms27156848)
Supplement: Supplementary file 1 [file ijms-27-06848-s001.zip › miRNA library-supplementary tables.pdf]

Table S1. Data filtering and statistics on the situation of removing junctions of ovraies

Sample	clean_reads	high_quality	3'adapter_null	insert_null	5'adapter_contaminants	polyA	clean_tags
EO-1	13951201 (100%)	13806759 (98.9647%)	35245 (0.2553%)	14905 (0.1080%)	5127 (0.0371%)	187 (0.0014%)	13488546 (96.6838%)
EO-2	14561442 (100%)	14436163 (99.1397%)	19782 (0.1370%)	18822 (0.1304%)	4847 (0.0336%)	84 (0.0006%)	14205545 (97.5559%)
EO-3	14644480 (100%)	14476548 (98.8533%)	83432 (0.5763%)	28834 (0.1992%)	5361 (0.0370%)	152 (0.0010%)	14015764 (95.7068%)
AO-1	15438197 (100%)	15275688 (98.9474%)	45338 (0.2968%)	24281 (0.1590%)	4701 (0.0308%)	259 (0.0017%)	15048032 (97.4727%)
AO-2	9613022 (100%)	9515645 (98.9870%)	11811 (0.1241%)	8796 (0.0924%)	2083 (0.0219%)	49 (0.0005%)	9375385 (97.5280%)
AO-3	11823282 (100%)	11705473 (99.0036%)	12920 (0.1104%)	20985 (0.1793%)	8304 (0.0709%)	78 (0.0007%)	11567660 (97.8380%)

Note: EO(Estrus Ovray); AO(Abnormal Ovray), The following table is the same.

Table S2. Comparison of ovraies tag abundance statistics with those of non-coding RNAs in the ncgb database

Sample	clean_reads	high_quality	3'adapter_null	insert_null	5'adapter_contaminants	polyA	clean_tags
EO-1	13488546	128972 (0.96%)	601 (0.00%)	9886 (0.07%)	20059 (0.15%)	207083 (1.54%)	13121945 (97.28%)
EO-2	14205545	140788 (0.99%)	1127 (0.01%)	6212 (0.04%)	17300 (0.12%)	188944 (1.33%)	13851174 (97.51%)
EO-3	14015764	143558 (1.02%)	1062 (0.01%)	6488 (0.05%)	19975 (0.14%)	319904 (2.28%)	13524777 (96.50%)
AO-1	15048032	115659 (0.77%)	1124 (0.01%)	4597 (0.03%)	14237 (0.09%)	79327 (0.53%)	14833088 (98.57%)
AO-2	9375385	62958 (0.67%)	676 (0.01%)	4727 (0.05%)	8374 (0.09%)	53850 (0.57%)	9244800 (98.61%)
AO-3	11567660	61561 (0.53%)	1268 (0.01%)	3359 (0.03%)	10844 (0.09%)	198911 (1.72%)	11291717 (97.61%)

EO-3	14644480 (100%)	14476548 (98.8533%)	83432 (0.5763%)	28834 (0.1992%)	5361 (0.0370%)	152 (0.0010%)	14015764 (95.7068%)
AO-1	15438197 (100%)	15275688 (98.9474%)	45338 (0.2968%)	24281 (0.1590%)	4701 (0.0308%)	259 (0.0017%)	15048032 (97.4727%)
AO-2	9613022 (100%)	9515645 (98.9870%)	11811 (0.1241%)	8796 (0.0924%)	2083 (0.0219%)	49 (0.0005%)	9375385 (97.5280%)
AO-3	11823282 (100%)	11705473 (99.0036%)	12920 (0.1104%)	20985 (0.1793%)	8304 (0.0709%)	78 (0.0007%)	11567660 (97.8380%)

Table S3. Statistical results of tag abundance in the reference genome for ovraies

sample	total_abundance	match_abundance	other_abundance
EO-1	13488546	10660237 (79.03%)	2828309 (20.97%)
EO-2	14205545	10577869 (74.46%)	3627676 (25.54%)
EO-3	14015764	10970901 (78.28%)	3044863 (21.72%)
AO-1	15048032	11837327 (78.66%)	3210705 (21.34%)
AO-2	9375385	7282246 (77.67%)	2093139 (22.33%)
AO-3	11567660	8627974 (74.59%)	2939686 (25.41%)

Table S4. Statistical results of tag abundance in exons for ovraies

sample	total_abundance	match_abundance	other_abundance
EO-1	13488546	13405 (0.10%)	13475141 (99.90%)
EO-2	14205545	9949 (0.07%)	14195596 (99.93%)
EO-3	14015764	12758 (0.09%)	14003006 (99.91%)
AO-1	15048032	7467 (0.05%)	15040565 (99.95%)
AO-2	9375385	4603 (0.05%)	9370782 (99.95%)
AO-3	11567660	6532 (0.06%)	11561128 (99.94%)

Table S5. Number of known miRNAs identified in ovraies and statistics on tag abundance

sample	total_abundance	mirna_num	tags_unique	tags_abundance	ratio(%)
EO-1	13488546	542	8642	444733	3.3
EO-2	14205545	548	9407	606898	4.27
EO-3	14015764	563	8883	491927	3.51
AO-1	15048032	554	9100	554068	3.68
AO-2	9375385	506	7731	393657	4.2
AO-3	11567660	528	8528	553363	4.78

Table S6. Number of new miRNAs identified in ovraies and statistics on tag abundance

sample	total_abundance	mirna_num	tags_unique	tags_abundance	ratio(%)
EO-1	13488546	146	307	3159	0.02
EO-2	14205545	150	306	3342	0.02
EO-3	14015764	154	320	3725	0.03
AO-1	15048032	141	294	3690	0.02
AO-2	9375385	136	232	1932	0.02
AO-3	11567660	141	267	3079	0.03
